# COMPARISON OF ULTRASOUND- VS LANDMARK-GUIDED INJECTIONS FOR MUSCULOSKELETAL PAIN: AN UMBRELLA REVIEW

**DOI:** 10.2340/jrm.v56.40769

**Published:** 2024-08-26

**Authors:** Peng-Chieh SHEN, Ting-Yu LIN, Wei-Ting WU, Levent ÖZÇAKAR, Ke-Vin CHANG

**Affiliations:** 1 Department of Physical Medicine and Rehabilitation, Lo-Hsu Medical Foundation, Inc., Lotung Poh-Ai Hospital, Yilan City, Taiwan; 2 Department of Physical Medicine and Rehabilitation, National Taiwan University Hospital, College of Medicine, National Taiwan University, Taipei, Taiwan; 3 Department of Physical Medicine and Rehabilitation, National Taiwan University Hospital, Bei-Hu Branch, Taipei, Taiwan; 4 Department of Physical and Rehabilitation Medicine, Hacettepe University Medical School, Ankara, Turkey; 5 Center for Regional Anesthesia and Pain Medicine, Wang-Fang Hospital, Taipei Medical University, Taipei, Taiwan

**Keywords:** ultrasonography, anatomy, intervention, accuracy, symptom

## Abstract

**Objective:**

This umbrella review synthesizes systematic reviews and meta-analyses to reach a conclusion concerning the overall effectiveness of ultrasound-guided vs landmark-guided injections for treating musculoskeletal pain.

**Design:**

Umbrella review.

**Methods:**

PubMed, EMBASE, MEDLINE, and Web of Science were searched for relevant systematic reviews and meta-analyses from inception to March 2024. Critical appraisal, data extraction, and synthesis were performed in accordance with the criteria for conducting an umbrella review.

**Results:**

Seventeen articles, comprising 4 systematic reviews and 13 meta-analyses, were included. Using the AMSTAR2 instrument for quality assessment, 3 articles were rated as high quality, 1 as moderate, 7 as low, and 6 as critically low. Generally, ultrasound-guided injections were found to be more accurate than landmark-guided injections, particularly in the shoulder joint, though the results for pain relief and functional outcomes varied. Ultrasound guidance was notably effective for injections into the bicipital groove, wrist, hip, and knee – yielding greater accuracy and improved pain management. Both ultrasound-guided and landmark-guided techniques showed low incidence of adverse effects.

**Conclusion:**

This umbrella review offers an in-depth analysis of the comparative effectiveness of ultrasound-guided and landmark-guided injections across a range of musculoskeletal sites/conditions. The findings suggest that ultrasound-guided is a reliable method.

## LAY ABSTRACT

Injection and aspiration procedures in medicine traditionally rely on palpating anatomical landmarks. With the advancement of technology, ultrasound offers real-time needle visualization throughout the procedure. In our study, we investigated whether using ultrasound-guided injections is more effective than using landmark-guided injections for treating musculoskeletal pain. Through comprehensive literature review and analysis, we found that ultrasound significantly improved the accuracy of injections, especially in areas like the bicipital groove, wrist, hip, and knee, leading to better pain relief. Both ultrasound- and landmark-guided injections had few side effects. Ultrasound emerges as a promising tool for enhancing injection precision and patient outcomes in musculoskeletal pain management.

Injection and aspiration procedures are commonly utilized in the fields of orthopaedics, rehabilitation, rheumatology, and sports medicine, with the objective of alleviating pain and inflammation as well as increasing function (1). There are a variety of therapeutic agents used in these procedures, with corticosteroids, local anaesthetics, hyaluronic acid, and platelet-rich plasma being the most frequent ones (2). Corticosteroids are well known for their potent anti-inflammatory properties (2). Local anaesthetics are often combined with corticosteroids to provide immediate pain relief and potentially to assist in diagnostic evaluations (2). Hyaluronic acid functions by increasing the viscosity of synovial fluid, which helps to reduce stress on articular cartilage (2, 3). Platelet-rich plasma is used to promote healing by introducing growth factors directly to the site of tissue injury (2, 4).

Needles are often associated with patient dissatisfaction because of the pain and anxiety they cause. Traditionally, interventions have been performed by palpating anatomical landmarks to guide needle placement. However, without direct visualization of the needle’s pathway, there is the risk of injuring surrounding structures (5). Landmark-guided (LMG) injections are also challenging in patients with anatomical variations (5). Imaging modalities such as fluoroscopy, computed tomography (CT), magnetic resonance imaging (MRI), and ultrasound (US) are valuable tools for enhancing the precision and safety of medical procedures. While fluoroscopy and CT are particularly useful for procedures involving bone lesions and joints of the axial skeleton and pelvis, concerns regarding radiation exposure limit their use (6). MRI provides superior soft tissue visualization with multi-planar views (6), but it has limitations such as high cost, long procedure time, reduced portability, and limited availability (7).

Of note, US provides real-time visualization of the needle, offering direct guidance and feedback through-out the procedure (8). This capability significantly reduces the risk of inadvertent damage to surrounding structures such as vascular tissues, nerves, and tendons (8). Furthermore, US is characterized by its lack of radiation, non-invasiveness, and cost-effectiveness (9). Consequently, ultrasound-guided (USG) injections have gained popularity across healthcare settings, as a safer and more precise alternative to other modalities. This umbrella review aims to consolidate findings from existing systematic reviews and meta-analyses on the effectiveness of USG vs LMG injections at different sites. The goal is to offer a clear summary of the current evidence regarding the possible benefits of US guidance in various musculoskeletal conditions.

## METHODS

### Protocol registration

We carried out an umbrella review following the Preferred Reporting Items for Systematic Reviews and Meta-Analysis (PRISMA) guidelines. Our protocol was registered on Inplasy.com under the reference number INPLASY202450055.

### Search strategy

We conducted a thorough search of the literature using databases such as PubMed, EMBASE, MEDLINE, and Web of Science, covering the period from their inception to March 2024. The aim was to identify publications, irrespective of language, that explore the comparison between USG and LMG injections for treating musculoskeletal disorders. The keywords used for the literature search comprised “ultrasound, “ultrasonography”, “sonography”, “landmark”, “blind”, “anatomical”, “palpation”, “intra-articular”, “joints”, “tendon”, “bursa”, “ligament”, “muscle”, “pain”, “injections”, “administration”, “aspiration”, “review”, “systematic review”, and “meta-analysis”. The following search algorithm was employed; (“ultrasound” or “ultrasonography” or “sonography”) and (“landmark” or “blind” or “anatomical” or “palpation”) and (“intra-articular” or “joints” or “tendon” or “bursa” or “ligament” or “muscle” or “pain”) and (“injection” or “administration” or “aspiration”) and (“review” or “systematic review” or “meta-analysis”). Subsequently, 2 independent authors (P-CS and T-YL) screened the titles and abstracts of potentially relevant studies.

### Inclusion and exclusion criteria

This umbrella review specifically targeted systematic reviews and meta-analyses meeting the following inclusion criteria: investigations (1) comparing USG vs LMG injections, (2) involving patients diagnosed with musculoskeletal pain, and (3) focusing on either living human or cadaver studies. Exclusion criteria encompassed studies that (*i*) lacked a systematic literature search strategy, (*ii*) investigated non-musculoskeletal diseases, (*iii*) utilized imaging methods apart from US navigation, (*iv*) were devoid of a control group (solely using USG or LMG injections), or (*v*) were purely animal studies. Additionally, the review excluded the following categories; commentary, editorial, letter to the editor, thesis, conference proceeding, and research protocol.

### Article selection and data extraction

After the initial screening of titles and abstracts, two authors (P-CS and T-YL) independently assessed the full texts of potentially suitable publications. Any disagreements on including/excluding articles were resolved through discussions or by reaching out to the corresponding author. We recorded various key details from the extracted data, including the lead author, country of origin, year of publication, protocol registration status, number and types of studies included, databases used for the literature search, parameters measured (such as accuracy or clinical/functional outcomes), and reported efficacy.

### Quality assessment

The methodological quality assessment of the retrieved articles was conducted using the AMSTAR2 (A Measurement Tool to Assess Systematic Reviews) instrument (10), which comprises 16 evaluation criteria. Seven critical items were emphasized, including precedent protocols, comprehensive literature search, inclusion/exclusion criteria, risk of bias assessment, appropriate meta-analytic methods, data interpretation, and identification of publication bias. Each criterion was scored as either “yes”, “no”, or “partial yes.” Next, the studies were categorized into high, moderate, low, or critically low quality based on these assessments. This rigorous evaluation process was independently carried out by 2 authors (P-CS and T-YL).

### Data analysis

This umbrella review presents findings at the level of systematic reviews and meta-analyses. From the extracted data, we assessed factors such as injection accuracy, pain reduction, and functional improvement following USG and LMG injections. Quantitative analysis results, including statistical significance and confidence intervals, were drawn from the included meta-analyses. Details of the studies included in each eligible review are outlined in Table SI.

## RESULTS

### Literature search

**Fig. 1:**
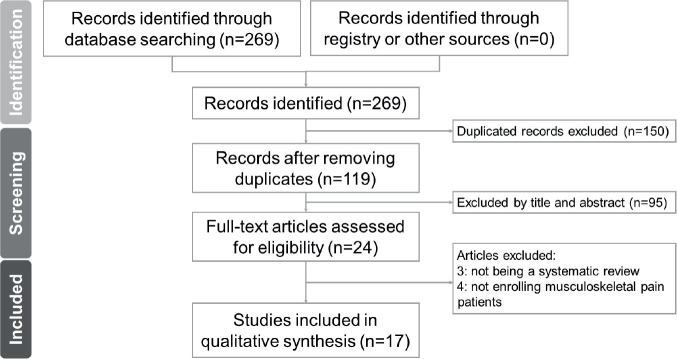
Flow diagram for the literature search.

Our comprehensive database search yielded a total of 269 records. Following the removal of duplicates, 119 records underwent initial screening based on their titles and abstracts. Subsequently, 24 papers were selected for a thorough full-text assessment. Among those, papers were excluded for not being a systematic review (*n* = 3) and not enrolling patients with musculoskeletal pain (*n* = 4). The details of article exclusion can be found in Tables SII and SIII. In total, 17 articles met the inclusion criteria and were analysed in the umbrella review (Fig. 1).

### Study characteristics

Table I summarizes the findings of the 17 reviews (11–27) that were published between 2011 and 2023. The number of studies included in each review ranged from 2 (11, 18) to 19 (27). The primary focus was on the shoulder joint, with further subdivisions into the glenohumeral joint (13, 18, 23, 27), subdeltoid/subacromial bursa (13, 15, 19, 21–23, 27), bicipital groove (13, 23), and non-differentiated shoulder area (11, 12, 23, 24, 26, 27). Two reviews were from Cochrane, with the initial edition released in 2012 by Bloom et al. (26), followed by an updated version in 2021 by Zadro et al. (27). Additionally, there were 2 reviews on the knee (17, 20), 1 on the hip (16), and 1 on the wrist – specifically addressing de Quervain disease injections (25). One review notably examined the efficacy of intra-articular and peri-articular injections in treating unspecified joint conditions (14).

**Table I:** Characteristics of the included reviews

Author, Year	Country	Protocol registration	Included studies (*n*)	Type of studies included	Searched database	Measurement parameters	Reported efficacy of US-guided injections
Shoulder–glenohumeral joint							
Aly et al., 2015	Canada	No	2	RCT	Cochrane, MEDLINE, PubMed	(1) Accuracy (2) VAS pain scale	USG>LMG in accuracy, VAS pain scale at 2 weeks post-injection USG ≒ LMG in VAS pain scale at 6 weeks post-injection
Simoni et al., 2017	Belgium	No	2	RCT	Cochrane, EMBASE, PubMed, Scopus	Accuracy of injection assessed by other medical imaging	USG>LMG in accuracy
Zadro et al., 2021	Australia	Cochrane review	3	RCT, quasi-RTC	Cochrane, EMBASE, MEDLINE	(1) Pain scores (VAS, NRS) (2) Function (SPADI, CMS, SDQ, DASH)	USG>LMG in pain score at 3–6 weeks post-injection USG ≒ LMG in function at 3–6 weeks post-injection
Fan et al., 2022	China	PROSPERO (CRD42021244790)	3	RCT	Cochrane, EMBASE, PubMed	(1) VAS pain scale (2) ASES score	USG ≒ LMG in VAS pain scale, ASES score at minimum follow-up period of 4 weeks (4–48 weeks) post-injection
Shoulder–subdeltoid/subacromial bursa							
Aly et al., 2015	Canada	No	6	RCT	Cochrane, MEDLINE, PubMed	(1) Accuracy (2) Pain score (VAS/NRS) (3) Function/disability (CMS, SPADI score, SDQ score, SF-36) (4) Adverse event	USG>LMG in pain score, function/disability at 6 weeks post-injection USG ≒ LMG in accuracy, adverse event at 6 weeks post-injection
Wu et al., 2015	China	No	7	RCT	Cochrane, EMBASE, Google Scholar, PubMed, Scopus, Web of Science	(1) VAS pain scale (2) Abduction degree (3) SDQ score (4) Function (SFA, CMS, physical function) (5) Effective rate	USG>LMG in VAS pain scale, SDQ score, abduction degree, function, effective rate at 6 weeks post-injection
Ayekoloye et al., 2020	USA	No	4	RCT	CINAHL, Cochrane, EMBASE, Google Scholar, PubMed, Scopus, Web of science	(1) VAS pain scale (2) SPADI pain score (3) SPADI disability score (4) SDQ score (5) Function (OSS, ASES, CMS)	USG>LMG in SPADI pain score at 4–6 weeks post-injection USG ≒ LMG in VAS pain scale, SDQ score, SPADI disability score, function at 4–6 weeks post-injection
Zadro et al., 2021	Australia	Cochrane review	12	RCT, quasi-RTC	Cochrane, EMBASE, MEDLINE	(1) Pain scores (VAS, NRS) (2) Function (SPADI, CMS, SDQ, DASH)	USG>LMG in pain score at 3–6 weeks post-injection USG ≒ LMG in function at 3–6 weeks post-injection
Adamson et al., 2022	UK	No	4	RCT	CINAHL, Cochrane, PubMed	(1) VAS pain scale (2) Function/disability (SPADI score, ASES score, CMS score, DASH questionnaire) (3) ROM (4) Adverse event	USG ≒ LMG in VAS pain scale, function/disability, ROM, adverse event 4–6 weeks post-injection
Fan et al., 2022	China	PROSPERO (CRD42021244790)	9	RCT	Cochrane, EMBASE, PubMed	(1) VAS pain scale (2) function/disability (ASES score, CMS score, SPADI score, SDQ score)	USG ≒ LMG in VAS pain scale, function/disability at minimum follow-up period of 4 weeks (4–48 weeks) post-injection
Deng et al., 2022	China	PROSPERO (CRD42020162682)	12	RCT	CBM, ClinicalTrials.gov, CNKI, Cochrane, EMBASE, PubMed, Scopus, Wanfang databases, Web of Science	(1) Pain scores (VAS, NRS, SPADI pain score) (2) Function/disability (SPADI disability score, CMS, SDQ, ASES, SFA, SF-36, patient global assessment, physicians global assessments) (3) ROM (4) Adverse event	USG>LMG in pain scores, function/disability at 6–8 weeks post-injection USG ≒ LMG in ROM, adverse event at 6–8 weeks post-injection
Shoulder–bicipital groove							
Aly et al., 2015	Canada	No	2	RCT	Cochrane, MEDLINE, PubMed	(1) Accuracy (2) VAS pain scale (3) Function (CMS)	USG>LMG in accuracy, VAS pain scale, function during procedure and at 4 weeks
Fan et al., 2022	China	PROSPERO (CRD42021244790)	2	RCT	Cochrane, EMBASE, PubMed	(1) VAS pain scale (2) Function (CMS)	USG>LMG in VAS pain scale, function at minimum follow-up period of 4 weeks (4–48 weeks) post-injection
**Shoulder injection without specifying sites**							
Soh et al., 2011	Singapore	No	2	RCT	Cochrane, EMBASE PubMed	(1) VAS pain scale (2) Function (SFA) (3) Adverse event	USG>LMG in VAS pain scale, function at 6 weeks post-injection (–) adverse event at 6 weeks post-injection
Bloom et al., 2012	Australia	Cochrane review	5	RCT, quasi-RTC	Cochrane, EMBASE, MEDLINE	(1) Pain scores (VAS, NRS) (2) Function (SPADI, CMS) (3) Abduction degree (4) Flexion degree (5) Adverse event	USG>LMG in pain scores at 6 weeks post-injection/ abduction degree at 2 weeks post-injection USG ≒ LMG in pain scores at 2 weeks post-injection/abduction degree, flexion degree, function, adverse event at 6 weeks post-injection
Sage et al., 2013	UK	No	6	RCT	AMED, EMBASE, PubMed	(1) VAS pain scale (2) Function (OSS, SFA) (3) Abduction degree (4) Flexion degree (5) Internal, external rotation degree	USG>LMG in VAS pain scale, abduction degree at 6 weeks post-injection USG ≒ LMG in function, flexion degree, internal, external rotation degree at 6 weeks post-injection
Zadro et al., 2021	Australia	Cochrane review	19	RCT, quasi-RTC	Cochrane, EMBASE, MEDLINE	(1) Pain scores (VAS, NRS) (2) Function (SPADI, CMS, SDQ, DASH) (3) Quality of life (4) Adverse event (5) Additional injections or surgery (6) Flexion degree (7) Abduction degree (8) External rotation degree	USG>LMG in pain score and abduction degree within 6 weeks post-injection, USG ≒ LMG in pain score and abduction degree over 6 weeks post-injection, function/quality of life, adverse event, additional injections or surgery, flexion degree, external rotation degree at any time post-injection
Fan et al., 2022	China	PROSPERO (CRD42021244790)	15	RCT	Cochrane, EMBASE, PubMed	(1) VAS pain scale (2) abduction degree (3) flexion degree (4) function (CMS score)	USG>LMG in abduction degree, flexion degree, function at minimum follow-up period of four weeks (4–48 weeks) post-injection USG ≒ LMG in VAS pain scale at minimum follow-up period of four weeks (4–48 weeks) post-injection
ElMeligie et al., 2023	Egypt	No	18	RCT	Cochrane, EBSCO, PubMed, Scopus, Web of Science	(1) VAS pain scale (2) Function (CMS, OSS, SFA, ASES) (3) Disability (DASH, SDQ, SPADI) (4) Abduction degree (5) Adverse event	USG>LMG in VAS pain score, function, abduction degree at 6 weeks post-injection USG ≒ LMG in disability, adverse event at 6 weeks post-injection
Wrist							
He et al., 2023	Canada	No	2	RCT	Cochrane, EMBASE MEDLINE	(1) VAS pain score (2) Symptom resolution rate	USG>LMG in VAS pain score, symptom resolution rates at 4 weeks post-injection
Hip							
Hoeber et al., 2016	USA	No	9	Uncontrolled trial	Cochrane, MEDLINE, PubMed	Accuracy of injection assessed by other medical imaging	USG>LMG in accuracy
Knee							
Wu et al., 2016	China	No	9	RCT, non-RCT	EMBASE, PubMed, Web of science	(1) Accuracy of injection assessed by other medical imaging (2) VAS pain scale (3) Aspiration volume (4) Procedure duration	USG>LMG in accuracy, VAS pain score at 2 weeks post-injection/ aspiration volume USG ≒ LMG in procedure duration
Fang et al., 2021	USA	No	12	RCT, uncontrolled trial	Cochrane, MEDLINE, PubMed	Accuracy of injection assessed by other medical imaging	USG>LMG in accuracy
Unspecified joints							
Huang et al., 2015	China	No	12	RCT	Cochrane, EMBASE, MEDLINE, Web of science	(1) VAS pain score (2) Accuracy of injection assessed by other medical imaging	USG>LMG in accuracy, VAS pain score at 2–6 weeks post-injection USG ≒ LMG in VAS pain score at 12 weeks

### Methodological quality of the included studies

Among the 17 reviews assessed, 3 were classified as high quality, 1 as moderate, 7 as low, and 6 as critically low (according to the AMSTAR2 system). Two of the reviews (22, 23) were registered with an international protocol registry (PROSPERO), whereas 2 others (26, 27) were from the Cochrane Database of Systematic Reviews. Most reviews (11–17, 19–21, 23, 25) did not provide a comprehensive list for excluding specific studies, and 6 reviews (12, 13, 16–18, 25) did not elucidate their criteria for selecting study designs for inclusion in their review. Detailed information regarding the methodological quality of the included reviews can be found in Table II.

**Table II:** Results of the AMSTAR-2 assessment

AMSTAR-2 item number																	
Author, year	1	2	3	4	5	6	7	8	9	10	11	12	13	14	15	16	Overall
Shoulder–glenohumeral joint																	
Aly et al., 2015	Y	PY	N	Y	Y	N	N	Y	Y	Y	Y	Y	Y	Y	N	N	CL
Simoni et al., 2017	Y	PY	N	Y	Y	Y	PY	N	N	Y	N/A	N/A	Y	Y	N/A	Y	L
Zadro et al., 2021	Y	Y	Y	Y	Y	Y	Y	Y	Y	Y	Y	Y	Y	Y	Y	Y	H
Fan et al., 2022	Y	Y	Y	Y	Y	Y	N	Y	Y	Y	Y	Y	Y	Y	Y	Y	L
Shoulder–subdeltoid/subacromial bursa																	
Aly et al., 2015	Y	PY	N	Y	Y	N	N	Y	Y	Y	Y	Y	Y	Y	N	N	CL
Wu et al., 2015	Y	PY	Y	PY	Y	Y	N	PY	Y	Y	Y	Y	Y	N	Y	N	L
Ayekoloye et al., 2020	Y	PY	Y	PY	Y	Y	N	PY	Y	Y	Y	Y	Y	Y	Y	Y	L
Zadro et al., 2021	Y	Y	Y	Y	Y	Y	Y	Y	Y	Y	Y	Y	Y	Y	Y	Y	H
Adamson et al., 2022	Y	PY	Y	PY	N	Y	N	Y	Y	Y	N/A	N/A	Y	Y	N/A	Y	L
Fan et al., 2022	Y	Y	Y	Y	Y	Y	N	Y	Y	Y	Y	Y	Y	Y	Y	Y	L
Deng et al., 2022	Y	Y	Y	Y	Y	Y	PY	Y	Y	Y	Y	Y	Y	Y	Y	Y	H
Shoulder–bicipital groove																	
Aly et al., 2015	Y	PY	N	Y	Y	N	N	Y	Y	Y	Y	Y	Y	Y	N	N	CL
Fan et al., 2022	Y	Y	Y	Y	Y	Y	N	Y	Y	Y	Y	Y	Y	Y	Y	Y	L
Shoulder injection without specifying sites																	
Soh et al., 2011	Y	N	Y	N	Y	Y	N	PY	Y	Y	Y	Y	Y	Y	N	N	CL
Bloom et al., 2012	Y	Y	Y	Y	Y	Y	Y	Y	Y	Y	Y	Y	Y	Y	Y	Y	H
Sage et al., 2013	Y	PY	N	PY	Y	Y	N	Y	N	Y	Y	N	N	N	N	Y	CL
Zadro et al., 2021	Y	Y	Y	Y	Y	Y	Y	Y	Y	Y	Y	Y	Y	Y	Y	Y	H
Fan et al., 2022	Y	Y	Y	Y	Y	Y	N	Y	Y	Y	Y	Y	Y	Y	Y	Y	L
ElMeligie et al., 2022	Y	PY	Y	PY	N	N	PY	PY	Y	Y	Y	Y	Y	Y	Y	Y	M
Wrist																	
He et al., 2023	Y	N	N	PY	Y	N	N	PY	N	Y	N/A	N/A	N	Y	N/A	Y	CL
Hip																	
Hoeber et al., 2016	Y	PY	N	Y	Y	N	N	PY	Y	Y	Y	Y	Y	N	N	N	CL
Knee																	
Wu et al., 2015	Y	PY	N	PY	Y	Y	N	PY	Y	Y	Y	Y	Y	N	Y	Y	L
Fang et al., 2021	Y	PY	Y	Y	Y	N	N	Y	Y	Y	N/A	N/A	Y	Y	N/A	Y	L
Unspecified joints																	
Huang et al., 2015	Y	N	Y	PY	Y	Y	N	Y	N	Y	Y	N	N	Y	N	Y	CL

Y: yes; N: no; PY: partial yes; N/A: not applicable due to absence of meta-analyses; H: high; M: moderate; L: low; CL: critically low; 1: PICO elements; 2: prior protocol; 3: study designs; 4: search strategy; 5: study selection; 6: data extraction; 7: excluded studies; 8: PICO details; 9: risk of bias assessment; 10: funding sources; 11: meta-analysis methods; 12: risk of bias impact on results; 13: risk of bias discussion; 14: explain heterogeneity; 15: publication bias; 16: conflict of interest.

### Summary of the outcome – shoulder

*Glenohumeral joint injection: Accuracy.* There were 2 articles focusing on this topic. Simoni et al. (18) conducted a systematic review to assess the accuracy of injections, involving a total of 100 patients and 80 cadavers. Various tools, such as fluoroscopy, MRI, dye location during dissection, and arthroscopy were employed. Their findings suggested that USG injections generally displayed higher accuracy compared with LMG injections in the glenohumeral joint (86% to 100% vs 45% to 100%). Another meta-analysis by Aly et al. (13) corroborated this finding (92.5% vs 72.5%), using fluoroscopy as the accuracy assessment. However, it was noted that certain LMG injections could achieve 100% accuracy.

*Pain.* Fan et al. (23) conducted a meta-analysis focusing on pain reduction following USG or LMG injections. USG injections did not show superiority over LMG in pain relief, measured by the visual analogue scale (VAS) score (weight mean differences [WMD]: –0.01 cm, 95% CI: –0.19 to 0.17, *p* = 0.78) during a minimum follow-up period of 4 (ranging from 4–48) weeks post-injection. Aly et al. (13) demonstrated that USG injections resulted in a greater reduction in pain (WMD: –0.6 cm, 95% CI: –0.8 to –0.4, *p* < 0.001) at the first 2 weeks after the intervention, with no significant difference observed between groups (WMD: 0.12 cm, 95% CI: –1.33 to 1.58, *p* = 0.87) at 6 weeks post-injection. The most recent Cochrane review, released in 2021, indicated a slight improvement in pain scores, with a reduction of 0.21 points (WMD: –0.21 points, 95% CI: –0.39 to –0.03, *p* = 0.02), favouring USG injections over LMG injections at 3–6 weeks post-injection (27).

*Function.* Fan et al.’s meta-analysis found that USG injections did not demonstrate superiority over LMG in improving functionality, as assessed by the American Shoulder and Elbow Surgeons Assessment Form score (WMD: –1.96 points, 95% CI: –7.41 to 3.49, *p* = 0.48) within 4–48 weeks follow-up post-injection. According to the Cochrane review in 2021 (27), there is evidence of moderate certainty, suggesting that USG injections may not lead to a notable improvement in function between 3–6 weeks post-injection, compared with injections without image guidance (standardized mean difference [SMD]: 1.05, 95% CI: –1.18 to 3.29, *p* = 0.35).

*Subdeltoid/subacromial bursa injection: Accuracy.* Aly et al. (13) found comparable accuracy levels between USG and LMG injections for the subacromial space using MR arthrography as the gold standard (65% vs 70%).

*Pain.* Four of the reviews (13, 15, 22, 27) indicated superior pain relief following USG injections, while the remaining reviews (21, 23) showed no significant difference between US vs landmark guidance. In the meta-analysis by Ayekoloye et al. (19), USG injections resulted in a greater reduction in the Shoulder Pain and Disability Index (SPADI) pain scores (WMD: 1.97 points, 95% CI: 0.35 to 3.58, *p* = 0.02) but not in VAS scores (WMD: –0.18 cm, 95% CI: –1.01 to 0.65, *p* = 0.67). The latest Cochrane review, involving 777 participants, concluded that USG injections yielded only a little improvement in pain scores at 3–6 weeks post-injection (27). Specifically, the mean pain score with LMG injections was 3.1 points, whereas USG injections showed a 0.6-point improvement (WMD: 0.6 points, 95% CI: 0.1 to 1.05, *p* = 0.02).

*Function.* Two early meta-analyses (13, 15) initially showed promising results favouring USG injections for shoulder function improvement. Wu et al. (15) assessed 4 RCTs and reported that USG injections were associated with increased shoulder function (SMD: 32.69, 95% CI: 14.82 to 50.56, *p* < 0.01) and decreased shoulder disability questionnaire scores (WMD: 5.01 points, 95% CI: 1.82 to 8.19, *p* = 0.02) compared with LMG injections. Similarly, Aly et al. (13) analysed 3 RCTs and reported similar results favouring US guidance at 6 weeks post-injection (SMD: 0.70, 95% CI: 0.39 to 1.01, *p* < 0.01). However, subsequent reviews including those by Ayekoloye et al. (19), Adamson et al. (21) and Fan et al. (23) did not observe any significant difference in various functional evaluations between the USG and LMG groups. The most recent systematic review and meta-analysis by Deng et al. (22) included 11 RCTs with a total of 851 participants and showed a small but significant improvement in function with USG injections (SMD: –0.84, 95% CI: –1.41 to –0.27, *p* = 0.004). However, this evidence was of very low certainty due to high heterogeneity (I^2^ = 92.8%). In the latest Cochrane review involving 687 participants, there is no evidence confirming an advantage of USG injections over LMG injections for improving function in subacromial impingement (SMD: 5.06, 95% CI: –3.23 to 13.35, *p* = 0.23) (27).

*Range of motion.* Regarding shoulder active range of motion, 2 reviews (21, 22) suggest that USG injections do not provide a significant advantage over LMG injections of the subacromial/subdeltoid bursa. However, 1 meta-analysis by Wu et al. (15) reported a slightly favourable outcome for the USG injections, specifically in the abduction degree (WMD: 0.89 degrees, 95% CI: 0.56 to 1.23, *p* < 0.01).

*Bicipital groove injection: Accuracy.* The assessment of accuracy was performed using CT in the review conducted by Aly et al. (13), whereby significantly higher accuracy rates were shown for USG vs LMG injections (86.7% vs 26.7%, *p* < 0.05).

*Pain.* In the review conducted by Aly et al. (13), it was observed that the USG group exhibited greater pain reduction compared with the LMG group (WMD: 1.9 cm, 95% CI: 1.2 to 2.6, *p* < 0.001) during the procedure and 4 weeks later. These findings were further supported by Fan et al. (23), who reported similar results, indicating that the USG group experienced less pain than the LMG injection group (WMD: 1.5 cm, 95% CI: 0.54 to 2.46, *p* = 0.02) at a minimum follow-up period of 4 weeks post-injection.

*Function.* Shoulder function was assessed using the Constant–Murley Shoulder Score. Aly et al. (13) reported notable improvement in function within the USG group (WMD: 10.9 points, 95% CI: 6.57 to 15.23, *p* < 0.00001). Similarly, Fan et al. (23) also observed comparable findings, indicating that the USG group achieved a better Constant–Murley Shoulder Score compared with the LMG group (WMD: 12.0 points, 95% CI: 5.74 to 18.26, *p* = 0.0002).

*Shoulder injection without specifying sites: Pain.* Six reviews, including 2 from Cochrane (26, 27) and 4 from Soh et al. (11), Sage et al. (12), Fan et al. (23), and ElMeligie et al. (24), provided information regarding shoulder injections overall, irrespective of pathology, location, or technique. Five reviews have favoured USG injections over LMG injections in terms of the mean change in VAS scores from baseline to 6 weeks. The sole exception was found in the meta-analysis by Fan et al. (23), which indicated no significant difference in VAS scores between the LMG and USG groups during a follow-up period ranging from 4 to 48 weeks post-injection.

In the initial 2012 version of the Cochrane review (26), although having considerable heterogeneity, there was significant difference favouring US guidance at 6 weeks post-injection (SMD: –0.80, 95% CI: –1.46 to –0.14, *p* = 0.017) (26). In the subsequent Cochrane review in 2021, which included 19 trials and 1,035 participants, USG injections were also associated with a slight improvement in pain up to 6 weeks post-injection (SMD: –0.52, 95% CI: –0.84 to –0.20, *p* = 0.002) compared with LMG injections (27). However, this subtle improvement was deemed unlikely to be clinically significant. Furthermore, no additional benefit was observed beyond 6 weeks post-injection (27).

*Function.* In the systematic review conducted by Soh et al. (11), 2 RCTs were analysed after strict exclusion criteria, i.e., pain duration of less than 3 weeks, prior shoulder trauma, or previous physiotherapy, and specifically targeted studies centred on single corticosteroid injections without prior local steroid administration. The findings indicated that patients who underwent USG injections experienced a significantly greater improvement in shoulder function at 6 weeks post-injection than with LMG injections (SMD: 1.09, 95% CI: 0.61 to 1.57, *p* < 0.01).

On the other hand, the reviews by Sage et al. (12) and Fan et al. (23) failed to identify any difference between USG vs LMG injections concerning shoulder function. The 2012 Cochrane review also concluded that USG injection did not offer any advantage over LMG injection in enhancing patients’ shoulder function within the initial 6-week period post-injection (SMD: 0.63, 95% CI: –0.06 to 1.33, *p* = 0.075) (26). Further, the 2021 Cochrane review reaffirmed this absence of benefits in functional improvement with USG injections within 6 months post-injection (SMD: 3.35, 95% CI: –4.69 to 11.38, *p* = 0.41) (27).

The latest review conducted by ElMeligie et al. (24) examined various functional assessment tools across 9 studies involving 482 patients, along with shoulder disability assessments from 6 studies comprising 342 patients. Their findings unveiled that USG injections yielded significantly better overall shoulder functional scores compared with LMG injections (SMD: 0.35, 95% CI: 0.05 to 0.65, *p* < 0.01). However, there was no significant distinction between the groups in terms of overall shoulder disability scores (SMD: –0.51, 95% CI: –1.25 to 0.22, *p* = 0.88).

*Range of motion.* Both Cochrane reviews (26, 27) and the review by Sage et al. (12) indicated that USG injection provided a negligible improvement in shoulder range of motion over LMG injection. The exception lies in a slight enhancement in shoulder abduction (WMD: 6.85 degrees, 95% CI: 1.47 to 12.22, *p* = 0.01) within 6 weeks post-injection, although supported by evidence with low certainty (27).

In a recent review, ElMeligie et al. (24) studied 18 RCTs (involving a total of 428 patients) and noted a similar increase in shoulder abduction degree in the USG vs LMG group (WMD: 8.78 degrees, 95% CI: 3.11 to 14.46, *p* < 0.01) at 6 weeks post-injection. Additionally, Fan et al. (23) demonstrated that patients receiving USG injections exhibited slightly superior abduction (WMD: 3.08 degrees, 95% CI: 0.98 to 5.19, *p* = 0.004) and flexion (WMD: 3.36 degrees, 95% CI: 1.16 to 1.56, *p* = 0.003) compared with the LMG group, in at least 4 weeks post-injection follow-up.

### Wrist

*Pain.* He et al. (25) undertook a systematic review on de Quervain disease and found 2 studies comparing USG and LMG techniques. Four weeks after treatment, pain scores were significantly lower in the USG (2.6 ± 1.5) vs LMG (5.8 ± 2.2) group. Additionally, the resolution rate, defined as the percentage of patients experiencing no pain and no disruption of daily life post-injection, significantly favoured the USG (95.7%) vs LMG (78.2%) group.

### Hip

*Accuracy.* A meta-analysis investigated the comparative accuracy between USG and LMG hip joint injections utilizing established gold standards, e.g., fluoroscopy, CT, MRI arthrography, or direct visualization of the injectate during surgery (16). The analysis revealed a significantly superior accuracy of USG procedures, being 100% (95% CI: 98% to 100%) as compared with that of LMG procedures, being 72% (95% CI: 56% to 85%).

### Knee

*Accuracy.* Two reviews compared the efficacy of USG and LMG knee injections/arthrocentesis. Fang et al. (20) gathered 12 studies and reported that USG procedures were more accurate than any LMG approach (including the mid-medial, mid-patellar, and suprapatellar bursae as well as the supralateral and superolateral portals). The studies used a range of methods to evaluate injection accuracy, e.g., post-injection radiographs evaluated by a blinded radiologist, assessment of arthrocentesis accuracy through the volume of fluid aspiration, contrast injection with fluoroscopy, monitoring solution diffusion with USG, mini air-arthrography, and the injection of methylene blue dye during arthroscopy followed by grading. The accuracies were at least 94% with USG from all portals. The most notable difference in accuracy between USG and LMG techniques was observed during mid-medial injections (97% vs 78%) and mid-patellar injections (95.6% vs 77.3%).

Wu et al. (17) compiled data from 8 studies involving arthrocentesis performed on 725 knee joints. The risk ratio of success for USG procedures was 1.21 (95% CI: 1.13 to 1.29, *p* < 0.001) in comparison with LMG techniques.

*Pain.* The procedure was significantly less painful during USG vs LMG knee effusion aspiration (WMD: –2.24 cm, 95% CI: –2.92 to –1.56, *p* < 0.001) (17). Furthermore, at 2-week follow-up, those receiving USG arthrocentesis achieved greater pain reduction (WMD: 0.84 cm, 95% CI: 0.42 to 1.27, *p* < 0.001) (17).

### Unspecified joints

*Accuracy.* In a systematic review and meta-analysis by Huang et al. (14), 12 RCTs comparing USG vs LMG injections or arthrocentesis for intra-articular and periarticular joints were assessed with the accuracy being determined using MRI and fluoroscopy. They found that US guidance substantially enhances the accuracy of various joint injections or arthrocentesis (odds ratio [OR]: 0.36, 95% CI: 0.22 to 0.60).

*Pain.* More significant decrease in VAS scores was noted up to 6 weeks post-injection by US guidance (WMD: –1.42 cm, 95% CI: –1.82 to –1.02, *p* < 0.001) (14). However, at the 12th week, there was no significant difference in VAS scores between USG and LMG intra- and peri-articular joint injections (WMD: –0.44, 95% CI: –1.17 to 0.28, *p* = 0.23).

### Adverse effects

Seven reviews (11, 13, 21, 22, 24, 26, 27) addressed the issue of adverse effects, including post-injection pain, skin peeling, facial redness, dizziness, and a feeling of post-injection warmth. Such adverse events were reported infrequently and were mild in both USG and LMG groups. The 2021 Cochrane review revealed that while there was a marginal reduction in adverse events associated with USG vs LMG injections (relative risk: 0.72, 95% CI: 0.4 to 1.28, with a 7% absolute difference), this difference did not reach statistical significance (*p* = 0.26) (27). Serious adverse events and withdrawals due to adverse events were seldom reported in either group (26, 27).

## DISCUSSION

**Fig. 2:**
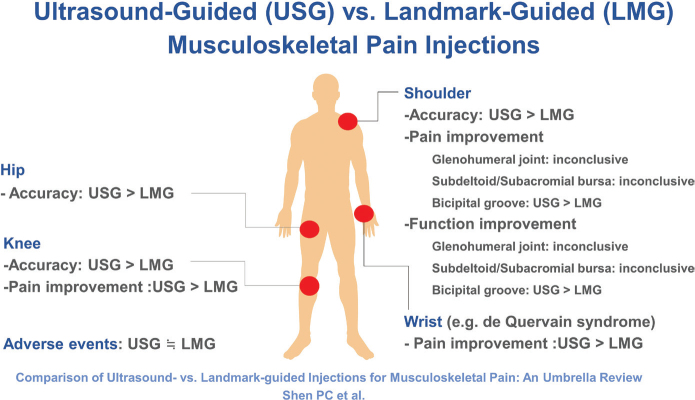
Findings summary of this umbrella review.

This umbrella review provides a comprehensive look into the current evidence comparing USG vs LMG injections at different sites/conditions. Concerning the shoulder, USG generally yields higher accuracy rates; however, conflicting findings exist as regards the efficacy of USG injections in pain alleviation and functional enhancement (Fig. 2). Notably, an exception lies in bicipital groove injections, where USG procedures demonstrate superior pain reduction and functional improvement compared with LMG injections. Regarding wrist injections (especially for de Quervain disease), US guidance yields significantly lower pain scores and higher resolution rates compared with LMG injections. In terms of the hip, USG interventions exhibit higher accuracy rates compared with LMG procedures. For knee injections, US guidance appeared more accurate and effective in reducing pain compared with landmark guidance. Overall, while USG injections are more accurate, it is unclear whether they offer greater benefits in terms of pain reduction or functional improvement. Both USG and LMG injections tend to have infrequent and mild adverse effects, with no significant differences in safety between the 2 approaches.

This umbrella review illustrates the first comprehensive synthesis concerning the role of US guidance across multiple injection sites/conditions. However, some limitations must be acknowledged. First, it included only a limited number of high-quality systematic reviews and meta-analyses with considerable heterogeneity, e.g., corticosteroid type/concentration/volume, patient blinding/positioning, injection technique, needle/syringe size, and follow-up duration. Second, it is constrained by the lack of comprehensive demographic data and details concerning adjuvant physiotherapy or medication, making it difficult to fully evaluate other factors that could influence treatment outcomes. Above all, the authors need to mention that the current/possible way of comparing pertinent data can actually be considered somewhat insufficient. This is true especially from the perspective of “US guidance” for musculoskeletal interventions (28). Similar to surgeons’ approach (who would not use their knives without seeing the site to be operated), interventional physicians need to examine the site of possible injection with USG in advance – before using their needles. In this way, USG naturally embraces the period of pre-intervention (decision-making), intervention (precise targeting), and post-intervention (prompt follow-up). Unless examining for the inevitably damaged structures during blind injections or altered decisions with the use of US examination, comparing blind LMG vs USG interventions – simply with pain/symptom relief – would be inadequate.

While the rationale for the use of USG for shoulder injections is that improved accuracy might lead to better clinical outcomes, the current evidence does not support this conclusively. In this respect, variability in pain outcomes may be influenced by a range of factors. Assessment of the individual papers within the previous meta-analysis indicated that open-label studies using USG and limiting their injection doses to 5 cc or less generally demonstrated better pain relief (29–32). In contrast, studies with double-blind designs and injections exceeding 5 cc found similar outcomes for both USG and LMG methods, suggesting that subjective pain assessment might introduce bias and that injection volume could affect symptom outcomes (33–35). When the volume is large enough, the injectate can be effectively diffused into adjacent structures even during LMG techniques. Systemic effects of steroids might play a role, as a randomized double-blind study showed similar short-term improvements in pain and disability from both US-guided subacromial and systemic gluteal corticosteroid injections in patients with rotator cuff disease (35). Furthermore, the expertise of the injector may also play a role. Articles favouring US guidance lack details regarding the experience of the injectors (30), whereas studies with similar outcomes involve experienced radiologists (33), surgeons (34), or physiatrists (36, 37) with over 10 years of experience in administering injections.

The diverse outcomes in function following shoulder injections can be attributed to several factors, the most important of which being heterogeneous evaluation tools. These meta-analyses pooled different measures that may not necessarily assess the exact same construct (12, 13, 15, 19, 21–24, 26, 27). Additionally, some reviews included patients with chronic symptoms (29, 38), potentially limiting the functional gains achievable solely through corticosteroid injection. This aspect poses a challenge in accurately assessing whether there exists a discernible difference between USG and LMG injections.

Some studies comparing USG and LMG injections in shoulder range of motion have shown improvements in abduction and flexion. However, the difference in abduction and flexion between USG and LMG injections was found to be less than 9 degrees (24). Previous systematic review and meta-analysis indicate that the minimal clinically important difference in shoulder range of motion after treatment is approximately 12 degrees (39), whereas a significant clinical benefit is typically considered to be around 30 degrees (40). Therefore, a difference of less than 9 degrees was deemed insufficient to provide additional clinical benefits for using USG injection.

Our umbrella review indicates that the disparity in accuracy between USG and LMG injections is especially noticeable when targeting the bicipital groove. The reason might be the inherent challenge in palpating the bicipital groove, situated deep within the shoulder and often obscured by the overlying deltoid muscle (41). This difficulty is further exacerbated in patients with obesity and muscularity (13, 41). With USG, we can inject more effectively into the bicipital groove, without delivering the drugs into the adjacent tissues and leading to better pain alleviation and functional improvement (13, 23).

Compared with LMG techniques, US guidance yields significantly lower pain scores and higher resolution rates for de Quervain disease. Direct visualization of the needle entering the tendon sheath presents a significant advantage, particularly given the high prevalence of anatomical variations in the first dorsal extensor compartment. Certain patients may exhibit a septum that divides the abductor pollicis longus and extensor pollicis brevis tendons into 2 distinct compartments (35.8–43.7%) (25, 42). Chang et al. (43) reported that the presence of an intra-compartment septum may lead to increased friction and subsequent tenosynovitis and that the patient might fail to improve despite repeat LMG injections (possibly being administered into the non-pathological compartment) (43). US guidance allows for more precise injections in patients with anatomical variations, which might not be achievable with LMG injections.

In the hip joint, USG injections consistently demonstrate higher accuracy rates in comparison with LMG injections. Conversely, LMG injections have been found to have lower accuracy rates, particularly among patients with advanced arthritis or elevated body mass index (16, 44). This suggests that USG procedures may offer improved precision and effectiveness, especially in challenging cases where LMG injections may be less reliable.

Regarding knee intra-articular injections, our umbrella review showed that US guidance is more accurate and effective at alleviating pain compared with LMG injections. USG injections demonstrated excellent accuracy (>95%) across all approaches in the knee. On the other hand, LMG injections demonstrated inconsistent accuracy, with the mid-patellar approach achieving a lower accuracy rate of 77.3% (34 out of 44) and the supralateral approach reaching a higher accuracy rate of 95.74% (45 out of 47) (20). In instances where LMG intra-articular knee injections are unsuccessful, the most common reason is inaccurate needle placement, whereby the injections are often delivered into the Hoffa’s fat pad (81% of cases) (17). Differences in injectors’ experience may also impact injection accuracy. Inexpert LMG injections had a high rate of missed injections, with only 79% (60 out of 76) hitting the target. In contrast, no significant differences in accuracy were observed between USG injections performed by inexpert vs expert physicians, at 94% (47 out of 50) and 94% (47 out of 50) respectively (20). In LMG aspiration of joint fluid, challenges can arise when the needle diameter is not suitable for extracting viscous joint fluid. US examination, however, allows the practitioner to evaluate the depth and viscosity of fluid collections, facilitating the selection of an appropriate needle for arthrocentesis (20). As a result, USG arthrocentesis and intra-articular knee injections offer better outcomes compared with LMG techniques. These benefits include greater accuracy in needle placement, lower pain scores during and after the procedure, and larger aspiration volumes (17, 20).

## CONCLUSIONS

This umbrella review demonstrates that USG injections generally provide greater accuracy and improved outcomes across various anatomical sites, compared with LMG injections. While USG shoulder injections offer higher accuracy, the impact on pain relief and functional improvement remains inconclusive. Notably, USG injections in the bicipital groove and wrist achieve superior pain management, functional outcome, and success rate. Additionally, USG injections for hip and knee joints consistently result in greater accuracy and enhanced pain reduction. Both USG and LMG interventions have a low incidence of mild adverse effects. Future research should comprise studies with robust methodology to solidify specific questions/findings and further explore the pros and cons of USG vs LMG injections.

## Supplementary Material

COMPARISON OF ULTRASOUND- VS LANDMARK-GUIDED INJECTIONS FOR MUSCULOSKELETAL PAIN: AN UMBRELLA REVIEW
