# Supplementary material for: COMPARISON OF ULTRASOUND- VS LANDMARK-GUIDED INJECTIONS FOR MUSCULOSKELETAL PAIN: AN UMBRELLA REVIEW
Source: J Rehabil Med. 2024 Aug 26;56:40769. doi: 10.2340/jrm.v56.40769 (PMC11367678; doi:10.2340/jrm.v56.40769)
Supplement: COMPARISON OF ULTRASOUND- VS LANDMARK-GUIDED INJECTIONS FOR MUSCULOSKELETAL PAIN: AN UMBRELLA REVIEW [file JRM-56-40769-s1.pdf]

Supplementary material has been published as submitted. It has not been copyedited, or typeset by Journal of Rehabilitation Medicine

Table SI. Original studies in the included reviews

**Author, year**	**Study design**	**USG participants**	**LMG participants**	**Injection administration & Dose**	**Location**	**Related reviews**
Balint et al., 2002 (1)	Non-RCT	32	32	Joint fluid aspiration	knee	Wu et al., 2016 (2)
Naredo et al., 2004 (3)	RCT	21	20	20 mg triamcinolone	shoulder (either the subacromial-subdeltoid bursa, biceps ten-don sheath or rotator cuL calcifications depending upon the ultrasound findings, or LMG injection into the subacromial space)	Soh et al. (4)<br>Bloom et al. (5)<br>Sage et al. (6)<br>Aly et al. (7)<br>Huang et al. (8)<br>Wu et al., 2015 (9)<br>ElMeligie et al. (10)<br>Zadro et al. (11)
Wind et al., 2004 (12)	Uncontrolled trial	NA	131	0.1 mL of methylene blue with 3 mL of normal saline	knee	Fang et al. (13)
Pourbagher et al., 2005 (14)	Uncontrolled trial	10	NA	2 mL of low-molecular-weight hyaluronan and 1 mL of nonionic contrast material	hip	Hoeber et al. (15)
Chen et al., 2006 (16)	RCT	20	20	1 mL of betamethasone and 1 mL of 1% lidocaine	shoulder (subdeltoid/subacromial bursa)	Bloom et al. (5)<br>Sage et al. (6)<br>Aly et al. (7)<br>Wu et al., 2015 (9)<br>Deng et al. (17)<br>ElMeligie et al. (10)

						Zadro et al. (11)
Rutten et al., 2007 (18)	RCT	10	10	1 mL methylprednisolone acetate at 40 mg/mL, 4 mL prilocaïnehydrochloride at 10 mg/mL and 0.02 mL (0.01 mmol) Gadolinium DTPA	shoulder (glenohumeral joint)	Huang et al. (8)<br>Simoni et al. (19)
Luz et al., 2008 (20)	RCT	30	30	1 mL of 2% lidocaine, 1.5 mL of triamcinolone hexacetonide, 0.5 mL of non-ionic contrast and 0.5 mL of air	wrist (with rheumatoid arthritis)	Huang et al. (8)
Toda et al., 2008 (21)	Uncontrolled trial	NA	80	2.5 mL hyaluronic acid and 2 mL of contrast material (iotrolan)	knee	Fang et al. (13)
Diraçoğlu et al., 2009 (22)	Uncontrolled trial	NA	16	2mL radio-opaque dye (iohexol 300mg/mL)	hip	Hoeber et al. (15)
Ekeberg et al., 2009 (23)	quasi RCT	53	53	2 mL (10 mg/mL) triamcinolone and 5 mL (10 mg/mL) lidocaine hydrochloride	shoulder (subdeltoid/subacromial bursa)	Bloom et al. (5)<br>Zadro et al. (11)
Im et al., 2009 (24)	RCT	45	44	2 mL of hyaluronic acid and 0.5 mL of contrast dye solution	knee	Huang et al. (8)<br>Wu et al., 2016 (2)<br>Fang et al. (13)
Lee et al., 2009 (25)	RCT	21	22	0.5 mL (20 mg) triamcinolone and 1.5 mL of 2% lidocaine and 3 mL of normal saline	shoulder (glenohumeral joint)	Bloom et al. (5)<br>Sage et al. (6)<br>Aly et al. (7)<br>Fan et al. (26)

						ElMeligie et al. (10)<br>Zadro et al. (11)
Sibbitt et al., 2009 (27)	RCT	74	74	triamcinolone acetonide sus pension (80 mg for the knee, shoulder, and hip, 60 mg for wrist and ankle, and 20 mg for small joints of the fingers) and 2%lidocaine (3 mL for large joints,1 mL for small joints)	intraarticular joint	Huang et al. (8)
Smith et al., 2009 (28)	Uncontrolled trial	28	NA	5 mL iodinated contrast agent (180 mg/mL iohexol)	hip	Hoeber et al. (15)
Ucuncu et al., 2009 (29)	RCT	30	30	1 mL 40 mg triamcinolone and 1 mL 1% lidocaine	shoulder (subdeltoid/subacromial bursa)	Soh et al. (4)<br>Bloom et al. (5)<br>Sage et al. (6)<br>Aly et al. (7)<br>Huang et al. (8)<br>Wu et al., 2015 (9)<br>Fan et al. (26)<br>ElMeligie et al. (10)<br>Zadro et al. (11)
Ziv et al., 2009 (30)	Uncontrolled trial	NA	40	10 mL of normal saline mixed with methylene blue dye	hip	Hoeber et al. (15)
Cunnington et al., 2010 (31)	RCT	92	92	40 mg of triamcinolone acetonide, 1 mL of 1% lidocaine, and 4 mL contrast agent iohexol (350 mg/mL	knee	Huang et al. (8)<br>Wu et al., 2016 (2)<br>Fang et al. (13)

				preparation; Omnipaque)		
Kurup et al., 2010 (32)	Uncontrolled trial	NA	40	Chirocaine (Levobupivacaine hydrochloride 2.5 mg, 10 mL) and corticosteroid injections (Depomedrone 80 mg)	hip	Hoeber et al. (15)
Panditaratne et al., 2010 (33)	RCT	41	17	80 mg of Depo-Medrol and 3–10 mL of bupivacaine or lidocaine	shoulder (subdeltoid/subacromial bursa)	Sage et al. (6)<br>Deng et al. (17)<br>ElMeligie et al. (10)
Wiler et al., 2010 (34)	RCT	39	27	Joint fluid aspiration	knee	Wu et al., 2016 (2)<br>Fang et al. (13)
Choudur et al., 2011 (35)	Uncontrolled trial	14	NA	12 mL diluted Gadolinium with saline (2.5 mL gadolinium in 250 mL saline)	hip	Hoeber et al. (15)
Curtiss et al., 2011 (36)	Single-blinded, prospective cadaveric study	20	20	colored liquid latex	knee	Wu et al., 2016 (2)
Hashiuchi et al., 2011 (37)	RCT	15	15	0.2 mL amidotrizoate sodium meglumine	shoulder (bicipital groove)	Aly et al. (7)<br>ElMeligie et al. (10)
Sibbitt et al., 2011 [1] (38)	RCT	124	120	1–5 mL (depending on joint size) of 1% lidocaine and 3 mL triamcinolone acetonide	joints with inflammatory arthritis	Huang et al. (8)
Sibbitt el al., 2011 [2] (39)	RCT	47	37	Joint fluid aspiration and injection of 80 mg of triamcinolone acetonide	knee	Huang et al. (8)<br>Wu et al., 2016 (2)<br>Fang et al. (13)

Zhang et al., 2011 (40)	RCT	53	45	1 mL triamcinolone and 1 mL lidocaine	shoulder (bicipital groove)	Aly et al. (7)<br>Fan et al. (26)<br>ElMeligie et al. (10)
Bum Park et al., 2012 (41)	RCT	50	49	2 mL hyaluronic acid (1%<br>; molecular weight, 940–1,020 kDa) and 0.5 mL contrast dye (ionporomide 0.6234 g/mL)	knee	Huang et al. (8)<br>Wu et al., 2016 (2)<br>Fang et al. (13)
Dogu et al., 2012 (42)	RCT	23	23	1 mL of 5 mg/mL betamethasone dipropionate, 9 mL of 10 mg/mL prilocaine hydrochloride and 0.02 mL of 0.01 mmol gadolinium diethylenetriaminepentaacetic acid	shoulder (subdeltoid/subacromial bursa)	Aly et al. (7)<br>Huang et al. (8)<br>Wu et al., 2015 (9)<br>Ayekoloye et al. (43)<br>Deng et al. (17)<br>Fan et al. (26)<br>ElMeligie et al. (10)<br>Zadro et al. (11)
Kume et al., 2012 (44)	RCT	22	22	20 mg of triamcinolone (40 mg/mL) and 1 mL of 1% lidocaine	wrist	He et al. (45)
Patel et al., 2012 (46)	RCT	40	40	3 mL of iohexol radiopaque contrast	shoulder (glenohumeral joint)	Aly et al. (7)<br>Simoni et al. (19)
Sibbitt et al., 2012 (47)	RCT	42	22	Joint fluid aspiration and injection of 80 mg triamcinolone acetonide suspension and 5 mL of 1% lidocaine	knee	Wu et al., 2016 (2)
Zufferey et al., 2012 (48)	RCT	32	33	2 mL of 7 mg of 1/3 soluble and 2/3	shoulder	Sage et al. (6)

				long-acting betamethasone	(subdeltoid/subacromial bursa)	Aly et al. (7)<br>Huang et al. (8)<br>Wu et al., 2015 (9)<br>Fan et al. (26)<br>ElMeligie et al. (10)<br>Zadro et al. (11)
Hsieh et al., 2013 (49)	RCT	48	48	0.5 mL dexamethasone suspension (5mg/mL) and 3 mL lidocaine (10 mg/mL)	shoulder (subdeltoid/subacromial bursa)	Aly et al. (7)<br>Wu et al., 2015 (9)<br>Ayekoloye et al. (43)<br>Deng et al. (17)<br>Fan et al. (26)<br>ElMeligie et al. (10)<br>Zadro et al. (11)
Jang et al., 2013 (50)	RCT	50	49	6 mL of a mixed material containing 1% lidocaine (1 mL) and triamcinolone 20 mg (1 mL) and nonionic contrast (4 mL)	knee	Wu et al., 2016 (2)<br>Fang et al. (13)
Kantarci et al., 2013 (51)	Uncontrolled trial	59	NA	dilute contrast material (0.5 mmol/l gadopentetate dimeglumine)	hip	Hoeber et al. (15)s
Mei-Dan et al., 2013 (52)	Uncontrolled trial	NA	55	20 mL air	hip	Hoeber et al. (15)
Park et al., 2013 (53)	RCT	120	NA	5mL mixed material consisting of 1% lidocaine (1 mL), 20 mg of triamcinolone (1 mL), and nonionic contrast (3 mL)	knee	Fang et al. (13)

Saeed et al., 2014 (54)	RCT	50	50	40 mg of methylprednisolone acetate with 4 mL of lidocaine	shoulder (subdeltoid/subacromial bursa)	Wu et al., 2015 (9)<br>Deng et al. (17)<br>ElMeligie et al. (10)<br>Zadro et al. (11)
Singh et al., 2014 (55)	Uncontrolled trial	NA	87	2.5mg, 10mLs of levobupivacaine hydrochloride and Depomedrone 80 mg	hip	Hoeber et al. (15)
Haghighat et al., 2016 (56)	RCT	20	20	40 mg methylprednisolone with 1 cc lidocaine 2%	shoulder (subdeltoid/subacromial bursa)	Ayekoloye et al. (43)<br>Adamson et al. (57)<br>Deng et al. (17)<br>Fan et al. (26)<br>ElMeligie et al. (10)<br>Zadro et al. (11)
Lee et al., 2015 (58)	RCT	38	39	40 mg triamcinolone acetonide and 2 mL of 2% lidocaine	shoulder (glenohumeral joint)	Fan et al. (26)<br>Zadro et al. (11)
Cole et al., 2016 (59)	RCT	28	28	1 mL of 40 mg/mL methylprednisolone acetate and 5 mL of 1% lidocaine hydrochloride	shoulder (subdeltoid/subacromial bursa)	Ayekoloye et al. (43)<br>Adamson et al. (57)<br>Deng et al. (17)<br>Fan et al. (26)<br>ElMeligie et al. (10)<br>Zadro et al. (11)
Hosseini et al., 2016 (60)	RCT	100	123	4mL hyaloronic acid and 1 mL of contrast media	knee	Fang et al. (13)
Bhayana et al., 2018 (61)	RCT	30	30	2 mL (40 mg/ mL)	shoulder	Adamson et al. (57)

				methylprednisolone and 2 mL of 1% lignocaine	(subdeltoid/subacromial bursa)	Deng et al. (17)<br>Fan et al. (26)<br>ElMeligie et al. (10)<br>Zadro et al. (11)
Kamal et al., 2018 (62)	RCT	25	25	6 mL of drug (5 mL of 0.25% bupivacaine and 40 mg methylprednisolone)	shoulder	Fan et al. (26)
Wada et al., 2018 (63)	RCT	75	75	2.5mL, 1% hyaluronic acid	knee	Fang et al. (13)
Chernchujit et al., 2019 (64)	RCT	66	66	10 mL volume of air	knee	Fang et al. (13)
Akbari et al., 2020 (65)	RCT	14	14	1 mL of 40 mg methylprednisolone and 4 mL procaine 2%	shoulder (subdeltoid/subacromial bursa)	Adamson et al. (57)<br>Deng et al. (17)<br>Fan et al. (26)s<br>ElMeligie et al. (10)<br>Zadro et al. (11)
Raeissadat et al., 2020 (66)	RCT	20	21	1 mL lidocaine 1%, then, 3 mL water soluble un-ionised contrast with 1 mL distilled water, and finally, 1 mL triamcinolone 40 mg/mL with 1 mL lidocaine 1%.	shoulder (glenohumeral joint)	ElMeligie et al. (10)<br>Zadro et al. (11)
Shin et al., 2020 (67)	RCT	22	22	0.5 mL triamcinolone acetonide (40 mg/mL)	wrist	He et al. (45)
Yiannakopoulos et al., 2020 (68)	RCT	22	22	1 mL triamcinolone acetate (40 mg/mL) and 9 mL bupivacaine 0.5%	Shoulder (bicipital groove)	Fan et al. (26)<br>ElMeligie et al. (10)

Azadvari et al., 2021 (69)	RCT	15	15	1 mL of methylprednisolone and 2 mL of lidocaine	shoulder (subdeltoid/subacromial bursa)	Deng et al. (17)<br>Fan et al. (26)<br>Zadro et al. (11)
Cho et al., 2021 (70)	RCT	45	45	40 mg of triamcinolone acetonide, 4 mL of 1% lido-<br>caine, 4 mL of normal saline, and 3 mL of water soluble unionised contrast	shoulder (glenohumeral joint)	Fan et al. (26)<br>ElMeligie et al. (10)<br>Zadro et al. (11)
Roddy et al., 2021 (71)	RCT	128	128	40 mg methylprednisolone and 1 mL of 1% lidocaine	shoulder (subdeltoid/subacromial bursa)	Deng et al. (17)<br>Zadro et al. (11)

Table SII. Keywords and search results in different databases

Database	Keyword	Filter	Date	Results
PubMed	(“ultrasound” or “ultrasonography” or “sonography”) AND (“landmark” or “blind” or “anatomical” or “palpation”) AND (“intra-articular” or “joints” or “tendon” or “bursa” or “ligament” or “muscle” or “pain”) AND (“injections” or “administration” or “aspiration”) AND (“review” or “systematic review” or “meta-analysis”)	Title Abstract	2024/3/31	65
Embase	(“ultrasound” or “ultrasonography” or “sonography”) AND (“landmark” or “blind” or “anatomical” or “palpation”) AND (“intra-articular” or “joints” or “tendon” or “bursa” or “ligament” or “muscle” or “pain”) AND (“injections” or “administration” or “aspiration”) AND (“review” or “systematic review” or “meta-analysis”)	Title Abstract	2024/3/31	110
Medline	(“ultrasound” or “ultrasonography” or “sonography”) AND (“landmark” or “blind” or “anatomical” or “palpation”) AND (“intra-articular” or “joints” or “tendon” or “bursa” or “ligament” or “muscle” or “pain”) AND (“injections” or “administration” or “aspiration”) AND (“review” or “systematic review” or “meta-analysis”)	Abstract	2024/3/31	48
Web of Science	(“ultrasound” or “ultrasonography” or “sonography”) AND (“landmark” or “blind” or “anatomical” or “palpation”) AND (“intra-articular” or “joints” or “tendon” or “bursa” or “ligament” or “muscle” or “pain”) AND (“injections” or “administration” or “aspiration”) AND (“review” or “systematic review” or “meta-analysis”)	Abstract	2024/3/31	46

Table SIII. Excluded studies and reasons

Reasons	Reference
Not conducting a systematic literature search	(72-74)
Not recruiting patients with musculoskeletal pain	(75-78)

Reference:

1. Balint PV, Kane D, Hunter J, McInnes IB, Field M, Sturrock RD. Ultrasound guided versus conventional joint and soft tissue fluid aspiration in rheumatology practice: a pilot study. J Rheumatol 2002; 29: 2209-2213.
2. Wu T, Dong Y, Song H, Fu Y, Li JH. Ultrasound-guided versus landmark in knee arthrocentesis: A systematic review. Semin Arthritis Rheum 2016; 45: 627-632. DOI: 10.1016/j.semarthrit.2015.10.011.
3. Naredo E, Cabero F, Beneyto P, Cruz A, Mondéjar B, Uson J, et al. A randomized comparative study of short term response to blind injection versus sonographic-guided injection of local corticosteroids in patients with painful shoulder. J Rheumatol 2004; 31: 308-314.
4. Soh E, Li W, Ong KO, Chen W, Bautista D. Image-guided versus blind corticosteroid injections in adults with shoulder pain: a systematic review. BMC Musculoskelet Disord 2011; 12: 137. DOI: 10.1186/1471-2474-12-137.
5. Bloom JE, Rischin A, Johnston RV, Buchbinder R. Image-guided versus blind glucocorticoid injection for shoulder pain. Cochrane Database Syst Rev 2012; 10.1002/14651858.CD009147.pub2: Cd009147. DOI: 10.1002/14651858.CD009147.pub2.
6. Sage W, Pickup L, Smith TO, Denton ER, Toms AP. The clinical and functional outcomes of ultrasound-guided vs landmark-guided injections for adults with shoulder pathology--a systematic review and meta-analysis. Rheumatology (Oxford) 2013; 52: 743-751. DOI: 10.1093/rheumatology/kes302.
7. Aly AR, Rajasekaran S, Ashworth N. Ultrasound-guided shoulder girdle injections are more accurate and more effective than landmark-guided injections: a systematic review and meta-analysis. Br J Sports Med 2015; 49: 1042-1049. DOI: 10.1136/bjsports-2014-093573.
8. Huang Z, Du S, Qi Y, Chen G, Yan W. Effectiveness of Ultrasound Guidance on Intraarticular and Periarticular Joint Injections: Systematic Review and Meta-analysis of Randomized Trials. Am J Phys Med Rehabil 2015; 94: 775-783. DOI: 10.1097/PHM.0000000000000260.
9. Wu T, Song HX, Dong Y, Li JH. Ultrasound-guided versus blind subacromial-subdeltoid bursa injection in adults with shoulder pain: A systematic review and meta-analysis. Semin Arthritis Rheum 2015; 45: 374-378. DOI: 10.1016/j.semarthrit.2015.05.011.
10. ElMeligie MM, Allam NM, Yehia RM, Ashour AA. Systematic review and meta-analysis on the effectiveness of ultrasound-guided versus landmark corticosteroid injection in the treatment of shoulder pain: an update. J Ultrasound 2023; 26: 593-604. DOI: 10.1007/s40477-022-00684-1.

11. Zadro J, Rischin A, Johnston RV, Buchbinder R. Image-guided glucocorticoid injection versus injection without image guidance for shoulder pain. Cochrane Database Syst Rev 2021; 8: CD009147. DOI: 10.1002/14651858.CD009147.pub3.

12. Wind WM, Jr., Smolinski RJ. Reliability of common knee injection sites with low-volume injections. J Arthroplasty 2004; 19: 858-861. DOI: 10.1016/j.arth.2004.02.042.

13. Fang WH, Chen XT, Vangsness CT, Jr. Ultrasound-Guided Knee Injections Are More Accurate Than Blind Injections: A Systematic Review of Randomized Controlled Trials. Arthrosc Sports Med Rehabil 2021; 3: e1177-e1187. DOI: 10.1016/j.asmr.2021.01.028.

14. Pourbagher MA, Ozalay M, Pourbagher A. Accuracy and outcome of sonographically guided intra-articular sodium hyaluronate injections in patients with osteoarthritis of the hip. J Ultrasound Med 2005; 24: 1391-1395. DOI: 10.7863/jum.2005.24.10.1391.

15. Hoeber S, Aly AR, Ashworth N, Rajasekaran S. Ultrasound-guided hip joint injections are more accurate than landmark-guided injections: a systematic review and meta-analysis. Br J Sports Med 2016; 50: 392-396. DOI: 10.1136/bjsports-2014-094570.

16. Chen MJ, Lew HL, Hsu TC, Tsai WC, Lin WC, Tang SF, et al. Ultrasound-guided shoulder injections in the treatment of subacromial bursitis. Am J Phys Med Rehabil 2006; 85: 31-35. DOI: 10.1097/01.phm.0000184158.85689.5e.

17. Deng X, Zhu S, Li D, Luo Y, Zhang X, Tan Y, et al. Effectiveness of Ultrasound-Guided Versus Anatomic Landmark-Guided Corticosteroid Injection on Pain, Physical Function, and Safety in Patients With Subacromial Impingement Syndrome: A Systematic Review and Meta-analysis. Am J Phys Med Rehabil 2022; 101: 1087-1098. DOI: 10.1097/PHM.0000000000001940.

18. Rutten MJ, Maresch BJ, Jager GJ, de Waal Malefijt MC. Injection of the subacromial-subdeltoid bursa: blind or ultrasound-guided? Acta Orthop 2007; 78: 254-257. DOI: 10.1080/17453670710013762.

19. Simoni P, Grumolato M, Malaise O, Preziosi M, Pasleau F, de Lemos Esteves F. Are blind injections of gleno-humeral joint (GHJ) really less accurate imaging-guided injections? A narrative systematic review considering multiple anatomical approaches. Radiol Med 2017; 122: 656-675. DOI: 10.1007/s11547-017-0772-4.

20. Luz KR, Furtado RN, Nunes CC, Rosenfeld A, Fernandes AR, Natour J. Ultrasound-guided intra-articular injections in the wrist in patients with rheumatoid arthritis: a double-blind, randomised controlled study. Ann Rheum Dis 2008; 67: 1198-1200. DOI: 10.1136/ard.2007.084616.

21. Toda Y, Tsukimura N. A comparison of intra-articular hyaluronan injection accuracy rates between three approaches based on radiographic severity of knee osteoarthritis. Osteoarthritis Cartilage 2008; 16: 980-985. DOI: 10.1016/j.joca.2008.01.003.

22. Diraçoğlu D, Alptekin K, Dikici F, Balci HI, Ozçakar L, Aksoy C. Evaluation of needle positioning during blind intra-articular hip injections for osteoarthritis: fluoroscopy versus arthrography. Arch Phys Med Rehabil 2009; 90: 2112-2115. DOI: 10.1016/j.apmr.2009.08.137.

23. Ekeberg OM, Bautz-Holter E, Tveitå EK, Juel NG, Kvalheim S, Brox JI. Subacromial ultrasound guided or systemic steroid injection for rotator cuff disease: randomised double blind

study. Bmj 2009; 338: a3112. DOI: 10.1136/bmj.a3112.
24. Im SH, Lee SC, Park YB, Cho SR, Kim JC. Feasibility of sonography for intra-articular injections in the knee through a medial patellar portal. J Ultrasound Med 2009; 28: 1465-1470. DOI: 10.7863/jum.2009.28.11.1465.
25. Lee HJ, Lim KB, Kim DY, Lee KT. Randomized controlled trial for efficacy of intra-articular injection for adhesive capsulitis: ultrasonography-guided versus blind technique. Arch Phys Med Rehabil 2009; 90: 1997-2002. DOI: 10.1016/j.apmr.2009.07.025.
26. Fan D, Liu X, Ma J, Zhang S, Sun J, Li Y, et al. Ultrasound Guidance Is Not Superior in Subacromial Bursa and Intraarticular Injections but Superior in Bicipital Groove: A Meta-analysis of Randomized Controlled Trials. Arthroscopy 2022; 38: 1642-1657. DOI: 10.1016/j.arthro.2021.12.013.
27. Sibbitt WL, Jr., Peisajovich A, Michael AA, Park KS, Sibbitt RR, Band PA, et al. Does sonographic needle guidance affect the clinical outcome of intraarticular injections? J Rheumatol 2009; 36: 1892-1902. DOI: 10.3899/jrheum.090013.
28. Smith J, Hurdle MF, Weingarten TN. Accuracy of sonographically guided intra-articular injections in the native adult hip. J Ultrasound Med 2009; 28: 329-335. DOI: 10.7863/jum.2009.28.3.329.
29. Ucuncu F, Capkin E, Karkucak M, Ozden G, Cakirbay H, Tosun M, et al. A comparison of the effectiveness of landmark-guided injections and ultrasonography guided injections for shoulder pain. Clin J Pain 2009; 25: 786-789. DOI: 10.1097/AJP.0b013e3181acb0e4.
30. Ziv YB, Kardosh R, Debi R, Backstein D, Safir O, Kosashvili Y. An inexpensive and accurate method for hip injections without the use of imaging. J Clin Rheumatol 2009; 15: 103-105. DOI: 10.1097/RHU.0b013e318190fa20.
31. Cunnington J, Marshall N, Hide G, Bracewell C, Isaacs J, Platt P, et al. A randomized, double-blind, controlled study of ultrasound-guided corticosteroid injection into the joint of patients with inflammatory arthritis. Arthritis Rheum 2010; 62: 1862-1869. DOI: 10.1002/art.27448.
32. Kurup H, Ward P. Do we need radiological guidance for hip joint injections? Acta Orthop Belg 2010; 76: 205-207.
33. Panditaratne N, Wilkinson C, Groves C, Chandramohan M. Subacromial impingement syndrome: a prospective comparison of ultrasound-guided versus unguided injection techniques. Ultrasound 2010; 18: 176-181. DOI: 10.1258/ult.2010.010022.
34. Wiler JL, Costantino TG, Filippone L, Satz W. Comparison of ultrasound-guided and standard landmark techniques for knee arthrocentesis. J Emerg Med 2010; 39: 76-82. DOI: 10.1016/j.jemermed.2008.05.012.
35. Choudur HN, Ellins ML. Ultrasound-guided gadolinium joint injections for magnetic resonance arthrography. J Clin Ultrasound 2011; 39: 6-11. DOI: 10.1002/jcu.20753.
36. Curtiss HM, Finnoff JT, Peck E, Hollman J, Muir J, Smith J. Accuracy of ultrasound-guided and palpation-guided knee injections by an experienced and less-experienced injector using a superolateral approach: a cadaveric study. Pm r 2011; 3: 507-515. DOI: 10.1016/j.pmrj.2011.02.020.

37. Hashiuchi T, Sakurai G, Morimoto M, Komei T, Takakura Y, Tanaka Y. Accuracy of the biceps tendon sheath injection: ultrasound-guided or unguided injection? A randomized controlled trial. J Shoulder Elbow Surg 2011; 20: 1069-1073. DOI: 10.1016/j.jse.2011.04.004.

38. Sibbitt WL, Jr., Band PA, Chavez-Chiang NR, Delea SL, Norton HE, Bankhurst AD. A randomized controlled trial of the cost-effectiveness of ultrasound-guided intraarticular injection of inflammatory arthritis. J Rheumatol 2011; 38: 252-263. DOI: 10.3899/jrheum.100866.

39. Sibbitt WL, Jr., Band PA, Kettwich LG, Chavez-Chiang NR, Delea SL, Bankhurst AD. A randomized controlled trial evaluating the cost-effectiveness of sonographic guidance for intra-articular injection of the osteoarthritic knee. J Clin Rheumatol 2011; 17: 409-415. DOI: 10.1097/RHU.0b013e31823a49a4.

40. Zhang J, Ebraheim N, Lause GE. Ultrasound-guided injection for the biceps brachii tendinitis: results and experience. Ultrasound Med Biol 2011; 37: 729-733. DOI: 10.1016/j.ultrasmedbio.2011.02.014.

41. Bum Park Y, Ah Choi W, Kim YK, Chul Lee S, Hae Lee J. Accuracy of blind versus ultrasound-guided suprapatellar bursal injection. J Clin Ultrasound 2012; 40: 20-25. DOI: 10.1002/jcu.20890.

42. Dogu B, Yucel SD, Sag SY, Bankaoglu M, Kuran B. Blind or ultrasound-guided corticosteroid injections and short-term response in subacromial impingement syndrome: a randomized, double-blind, prospective study. Am J Phys Med Rehabil 2012; 91: 658-665. DOI: 10.1097/PHM.0b013e318255978a.

43. Ayekoloye CI, Nwangwu O. Ultrasound-Guided Versus Anatomic Landmark-Guided Steroid Injection of the Subacromial Bursa in the Management of Subacromial Impingement: A Systematic Review of Randomised Control Studies. Indian J Orthop 2020; 54: 10-19. DOI: 10.1007/s43465-020-00148-w.

44. Kume K, Amano K, Yamada S, Amano K, Kuwaba N, Ohta H. In de Quervain's with a separate EPB compartment, ultrasound-guided steroid injection is more effective than a clinical injection technique: a prospective open-label study. J Hand Surg Eur Vol 2012; 37: 523-527. DOI: 10.1177/1753193411427829.

45. He KS, He KS, Cheah A, Al-Halabi B, Danino MA, Efanov JI. Unblinding de Quervain: A systematic review of ultrasound-guided injection of corticosteroids for treatment of stenosing tenosynovitis of the 1st extensor compartment. J Med Radiat Sci 2023; 70: 319-326. DOI: 10.1002/jmrs.681.

46. Patel DN, Nayyar S, Hasan S, Khatib O, Sidash S, Jazrawi LM. Comparison of ultrasound-guided versus blind glenohumeral injections: a cadaveric study. J Shoulder Elbow Surg 2012; 21: 1664-1668. DOI: 10.1016/j.jse.2011.11.026.

47. Sibbitt WL, Jr., Kettwich LG, Band PA, Chavez-Chiang NR, DeLea SL, Haseler LJ, et al. Does ultrasound guidance improve the outcomes of arthrocentesis and corticosteroid injection of the knee? Scand J Rheumatol 2012; 41: 66-72. DOI: 10.3109/03009742.2011.599071.

48. Zufferey P, Revaz S, Degailler X, Balague F, So A. A controlled trial of the benefits of ultrasound-guided steroid injection for shoulder pain. Joint Bone Spine 2012; 79: 166-169. DOI: 10.1016/j.jbspin.2011.04.001.

49. Hsieh LF, Hsu WC, Lin YJ, Wu SH, Chang KC, Chang HL. Is ultrasound-guided injection more effective in chronic subacromial bursitis? Med Sci Sports Exerc 2013; 45: 2205-2213.

DOI: 10.1249/MSS.0b013e31829b183c.
50. Jang SH, Lee SC, Lee JH, Nam SH, Cho KR, Park Y. Comparison of ultrasound (US)-guided intra-articular injections by in-plain and out-of-plain on medial portal of the knee. Rheumatol Int 2013; 33: 1951-1959. DOI: 10.1007/s00296-012-2660-5.
51. Kantarci F, Ozbayrak M, Gulsen F, Gencturk M, Botanlioglu H, Mihmanli I. Ultrasound-guided injection for MR arthrography of the hip: comparison of two different techniques. Skeletal Radiol 2013; 42: 37-42. DOI: 10.1007/s00256-011-1306-0.
52. Mei-Dan O, McConkey MO, Petersen B, McCarty E, Moreira B, Young DA. The anterior approach for a non-image-guided intra-articular hip injection. Arthroscopy 2013; 29: 1025-1033. DOI: 10.1016/j.arthro.2013.02.014.
53. Park KD, Ahn JK, Lee SC, Lee J, Kim J, Park Y. Comparison of ultrasound-guided intra-articular injections by long axis in plane approach on three different sites of the knee. Am J Phys Med Rehabil 2013; 92: 990-998. DOI: 10.1097/PHM.0b013e3182923691.
54. Saeed A, Khan M, Morrissey S, Kane D, Fraser AD. Impact of outpatient clinic ultrasound imaging in the diagnosis and treatment for shoulder impingement: a randomized prospective study. Rheumatol Int 2014; 34: 503-509. DOI: 10.1007/s00296-013-2892-z.
55. Singh J, Khan WS, Marwah S, Wells G, Tannous DK, Sharma HK. Do we need radiological guidance for intra-articular hip injections? Open Orthop J 2014; 8: 114-117. DOI: 10.2174/1874325001408010114.
56. Haghighat S, Taheri P, Banimehdi M, Taghavi A. Effectiveness of Blind & Ultrasound Guided Corticosteroid Injection in Impingement Syndrome. Glob J Health Sci 2015; 8: 179-184. DOI: 10.5539/gjhs.v8n7p179.
57. Adamson NJ, Tsuro M, Adams N. Ultrasound-guided versus landmark-guided subacromial corticosteroid injections for rotator cuff related shoulder pain: A systematic review of randomised controlled trials. Musculoskeletal Care 2022; 20: 784-795. DOI: 10.1002/msc.1643.
58. Lee HJ, Ok JH, Park I, Bae SH, Kim SE, Shin DJ, et al. A Randomized Comparative Study of Blind versus Ultrasound Guided Glenohumeral Joint Injection of Corticosteroids for Treatment of Shoulder Stiffness. Clin Shoulder Elb 2015; 18: 120-127. DOI: 10.5397/cise.2015.18.3.120.
59. Cole BF, Peters KS, Hackett L, Murrell GA. Ultrasound-Guided Versus Blind Subacromial Corticosteroid Injections for Subacromial Impingement Syndrome: A Randomized, Double-Blind Clinical Trial. Am J Sports Med 2016; 44: 702-707. DOI: 10.1177/0363546515618653.
60. Hosseini B. Accuracy of Ultrasound Guided Versus Blind Knee Intra-articular Injection for Knee Osteoarthritis Prolotherapy. Journal of Anesthesia & Critical Care: Open Access 2016; 5. DOI: 10.15406/jaccoa.2016.05.00181.
61. Bhayana H, Mishra P, Tandon A, Pankaj A, Pandey R, Malhotra R. Ultrasound guided versus landmark guided corticosteroid injection in patients with rotator cuff syndrome: Randomised controlled trial. J Clin Orthop Trauma 2018; 9: S80-s85. DOI: 10.1016/j.jcot.2017.01.005.

62. Kamal K, Dahiya N, Singh R, Saini S, Taxak S, Kapoor S. Comparative study of anatomical landmark-guided versus ultrasound-guided suprascapular nerve block in chronic shoulder pain. Saudi J Anaesth 2018; 12: 22-27. DOI: 10.4103/sja.SJA_123_17.

63. Wada M, Fujii T, Inagaki Y, Nagano T, Tanaka Y. Isometric Contraction of the Quadriceps Improves the Accuracy of Intra-Articular Injections into the Knee Joint via the Superolateral Approach. JB JS Open Access 2018; 3: e0003. DOI: 10.2106/jbjs.Oa.18.00003.

64. Chernchujit B, Tharakulphan S, Apivatgaroon A, Prasetia R. Accuracy comparisons of intra-articular knee injection between the new modified anterolateral Approach and superolateral approach in patients with symptomatic knee osteoarthritis without effusion. Asia Pac J Sports Med Arthrosc Rehabil Technol 2019; 17: 1-4. DOI: 10.1016/j.asmart.2019.02.001.

65. Akbari N, Ozen S, Şenlikçi HB, Haberal M, Çetin N. Ultrasound-guided versus blind subacromial corticosteroid and local anesthetic injection in the treatment of subacromial impingement syndrome: A randomized study of efficacy. Jt Dis Relat Surg 2020; 31: 115-122. DOI: 10.5606/ehc.2020.71056.

66. Raeissadat SA, Nouri F, Darvish M, Esmaily H, Ghazihosseini P. Ultrasound-Guided Injection of High Molecular Weight Hyaluronic Acid versus Corticosteroid in Management of Plantar Fasciitis: A 24-Week Randomized Clinical Trial. J Pain Res 2020; 13: 109-121. DOI: 10.2147/jpr.S217419.

67. Shin YH, Choi SW, Kim JK. Prospective randomized comparison of ultrasonography-guided and blind corticosteroid injection for de Quervain's disease. Orthop Traumatol Surg Res 2020; 106: 301-306. DOI: 10.1016/j.otsr.2019.11.015.

68. Yiannakopoulos CK, Megaloikonomos PD, Foufa K, Gliatis J. Ultrasound-guided versus palpation-guided corticosteroid injections for tendinosis of the long head of the biceps: A randomized comparative study. Skeletal Radiol 2020; 49: 585-591. DOI: 10.1007/s00256-019-03315-9.

69. Azadvari M, Emami-Razavi SZ, Torfi F, Nazar NSB, Malekirad AA. Ultrasound-guided versus blind subacromial bursa corticosteroid injection for paraplegic spinal cord injury patients with rotator cuff tendinopathy: a randomized, single-blind clinical trial. Int J Neurosci 2021; 131: 445-452. DOI: 10.1080/00207454.2020.1748620.

70. Cho CH, Min BW, Bae KC, Lee KJ, Kim DH. A prospective double-blind randomized trial on ultrasound-guided versus blind intra-articular corticosteroid injections for primary frozen shoulder. Bone Joint J 2021; 103-b: 353-359. DOI: 10.1302/0301-620x.103b2.Bjj-2020-0755.R1.

71. Roddy E, Ogollah RO, Oppong R, Zwierska I, Datta P, Hall A, et al. Optimising outcomes of exercise and corticosteroid injection in patients with subacromial pain (impingement) syndrome: a factorial randomised trial. Br J Sports Med 2021; 55: 262-271. DOI: 10.1136/bjsports-2019-101268.

72. Perlas A, Wong P, Abdallah F, Hazrati LN, Tse C, Chan V. Ultrasound-guided popliteal block through a common paraneural sheath versus conventional injection: A prospective, randomized, double-blind study. Regional Anesthesia and Pain Medicine 2013; 38: 218-225. DOI: 10.1097/AAP.0b013e31828db12f.

73. Trainor D, Moeschler S, Pingree M, Hoelzer B, Wang Z, Mauck W, et al. Landmark-based versus ultrasound-guided ilioinguinal/iliohypogastric nerve blocks in the treatment of chronic postherniorrhaphy groin pain: a retrospective study. J Pain Res 2015; 8: 767-770. DOI: 10.2147/jpr.S86777.

74. Kim DH, Choi SS, Yoon SH, Lee SH, Seo DK, Lee IG, et al. Ultrasound-guided genicular nerve block for knee osteoarthritis: A double-blind, randomized controlled trial of local

anesthetic alone or in combination with corticosteroid. Pain Physician 2018; 21: 41-51.

75. Morgan PA, Monaghan GA, Richards S. A Systematic Review of Ultrasound-Guided and Non Ultrasound-Guided Therapeutic Injections to treat Morton's neuroma. J Am Podiatr Med Assoc 2014; 10.7547/12-124.1. DOI: 10.7547/12-124.1.

76. Koutsianas C, Klocke R. Efficacy of ultrasound-guided versus landmark-guided injections in rheumatology: A systematic review. Rheumatology (United Kingdom) 2017; 56: ii155. DOI: 10.1093/rheumatology/kex062.002.

77. Buntragulpoontawee M, Chang K-V, Vitoonpong T, Pornjaksawan S, Kitisak K, Saokaew S, et al. The Effectiveness and Safety of Commonly Used Injectates for Ultrasound-Guided Hydrodissection Treatment of Peripheral Nerve Entrapment Syndromes: A Systematic Review. Frontiers in pharmacology 2021; 11: 621150. DOI: 10.3389/fphar.2020.621150.

78. Grape S, Kirkham KR, Albrecht E. Transversus abdominis plane block versus local anaesthetic wound infiltration for analgesia after caesarean section A systematic review and meta-analysis with trial sequential analysis. EUROPEAN JOURNAL OF ANAESTHESIOLOGY 2022; 39: 244-251. DOI: 10.1097/EJA.0000000000001552.
